# Lacrimal Drainage System Obstruction Following Inferior Turbinate Surgery

**DOI:** 10.3389/fsurg.2020.590988

**Published:** 2020-10-15

**Authors:** Hirohiko Tachino, Hiromasa Takakura, Hideo Shojaku, Michiro Fujisaka, Chiharu Fuchizawa, Atsushi Hayashi

**Affiliations:** ^1^Department of Otolaryngology, Head and Neck Surgery, University of Toyama, Toyama, Japan; ^2^Head and Neck Surgery, Faculty of Medicine, Academic Assembly, University of Toyama, Toyama, Japan; ^3^Department of Ophthalmology, University of Toyama, Toyama, Japan

**Keywords:** nasolacrimal drainage system, location of obstruction, complication of inferior turbinate surgery, CT-dacrocystography, marsupialization

## Abstract

**Objective:** To provide information on the clinical characteristics and management of an uncommon acquired nasolacrimal drainage system obstruction (NLDSO).

**Methods:** A patient was treated with microdebrider-assisted endoscopic marsupialization for a cystic lesion located in the synechiae between the inferior turbinate and the lateral wall of the inferior meatus.

**Results:** A 21-year-old woman suffered from epiphora and purulent discharge 1 week after inferior turbinate surgery. Endoscopy reveled synechiae between the inferior turbinate and the lateral wall of the inferior meatus. Computed tomography-dacrocystography (CT-DCG) showed enlargement of the nasolacrimal duct and cystic accumulation of contrast medium from the lacrimal orifice posteriorly. The patient was treated with nasal endoscopic marsupialization of the cyst and then powered inferior turbinoplasty. Six months after the operation, both endoscopy and CT scan showed a widely patent left inferior meatus and nasolacrimal orifice.

**Conclusions:** We report the first case of post-operative NLDSO following inferior turbinate surgery. Nasal endoscopy and CT-DCG are useful to diagnose the location of a NLDSO. Otorhinolaryngologists should be aware that surgery may lead to the formation of a NLDSO and that endoscopic marsupialization is a curative treatment for these distal-end NLDSO. NLDSOs are caused by synechiae, which are a complication of inferior turbinate surgery.

## Introduction

Inferior turbinate hypertrophy is one of the main causes of chronic nasal obstruction and can have an allergic, infectious or vasomotoric etiology. Inferior turbinate surgery is widely performed to treat this disease. Complications of inferior turbinate surgery are rare (1), but bleeding and crusting are the most frequently described complications. Bone necrosis, synechiae, anosmia, atrophic rhinitis, and empty nose syndrome are other rare complications. We report the first case, to our knowledge, of post-operative nasolacrimal drainage system obstruction (NLDSO) following inferior turbinate surgery.

## Case Report

The patient was a 21-year-old female university student who had undergone bilateral submucosal resection of the inferior turbinate at another hospital to treat her nasal obstruction. She had never complained of epiphora before her first surgery. One week after discharge, she began to experience both epiphora from her left eye and swelling of her left eyelid, but her symptoms were tolerable. So, she left her symptoms untreated for 6 months until she began to suffer from purulent discharge from the left eye. She visited the same hospital again, where the doctor in charge suspected dacrycystitis and subsequently referred her to our hospital where her university was located.

**Figure 1:**
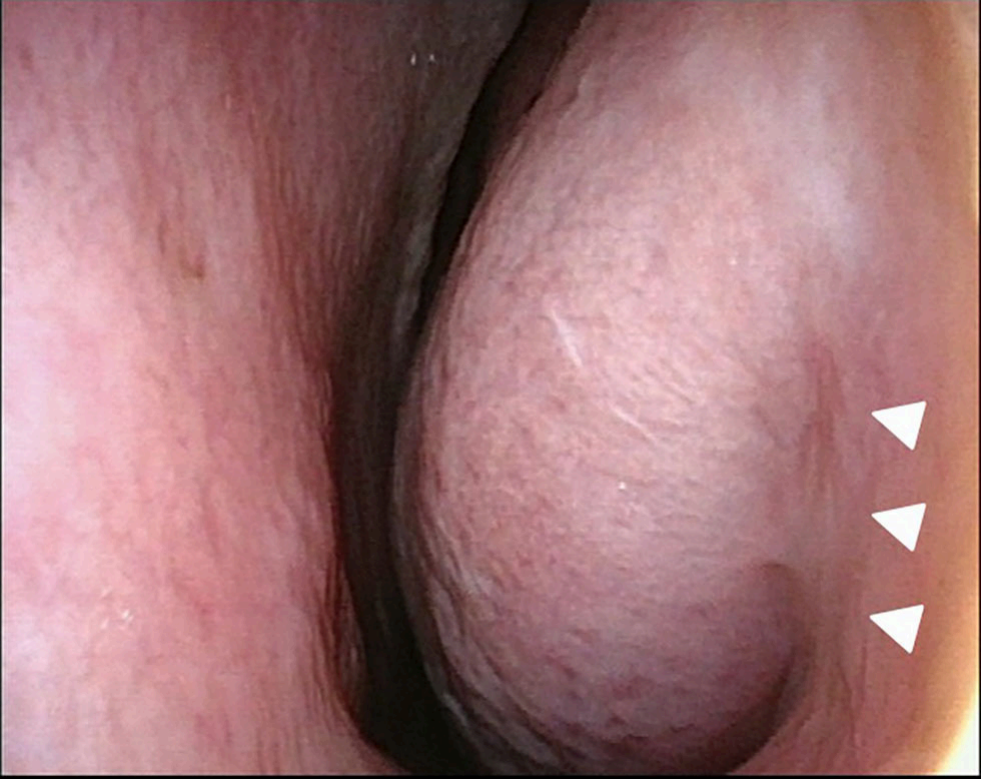
Flexible endonasal fiberscopic findings of the left nasal cavity. Hypertrophy of the left inferior turbinate was present, and the left inferior nasal meatus had disappeared (white arrowheads).

**Figure 2:**
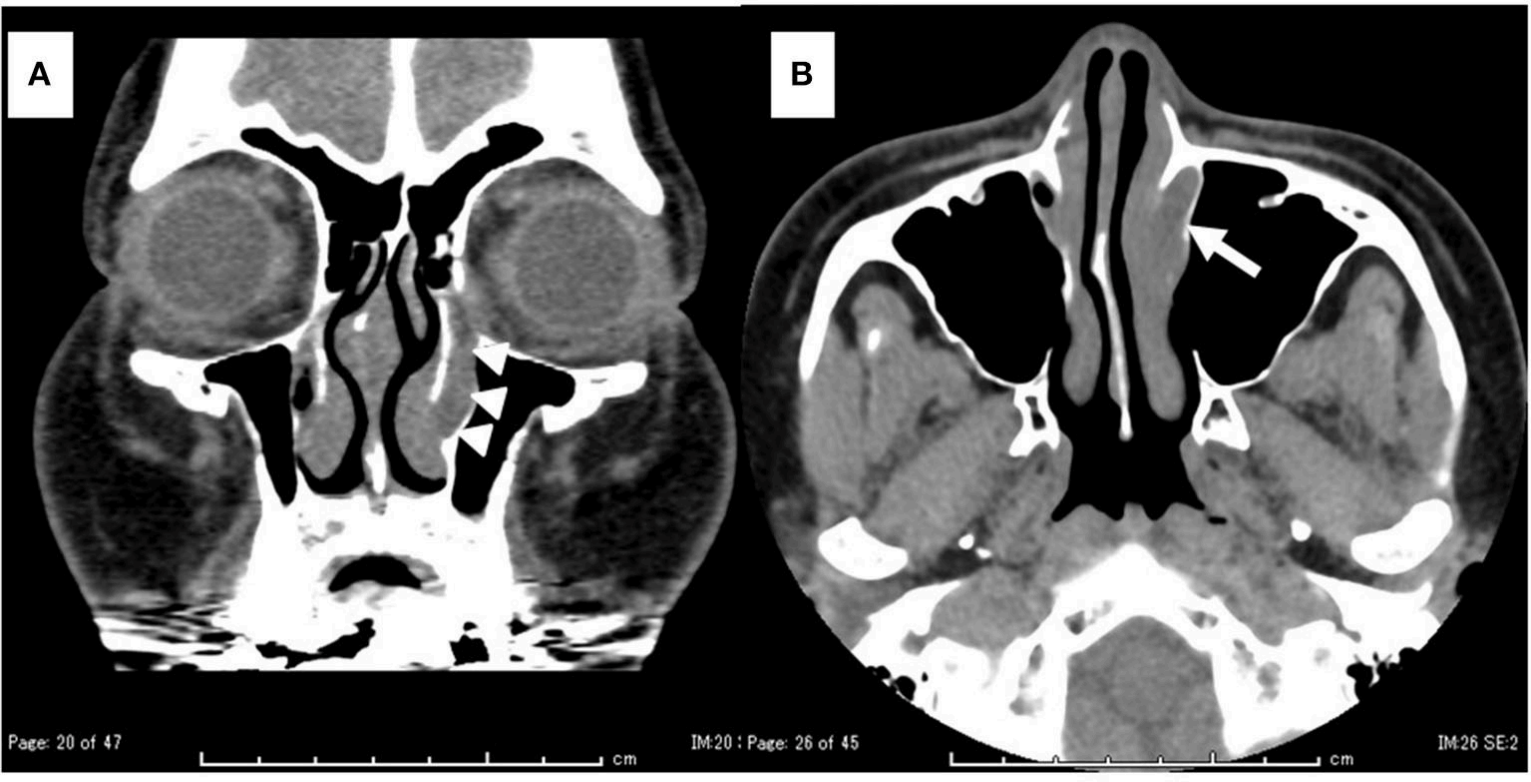
Computed tomography (CT) findings. **(A)** Coronal view: the left nasolacrimal duct was enlarged from the lacrimal sac to the orifice (white arrowhead). **(B)** Axial view: the left nasolacrimal orifice was enlarged (white arrow).

**Figure 3:**
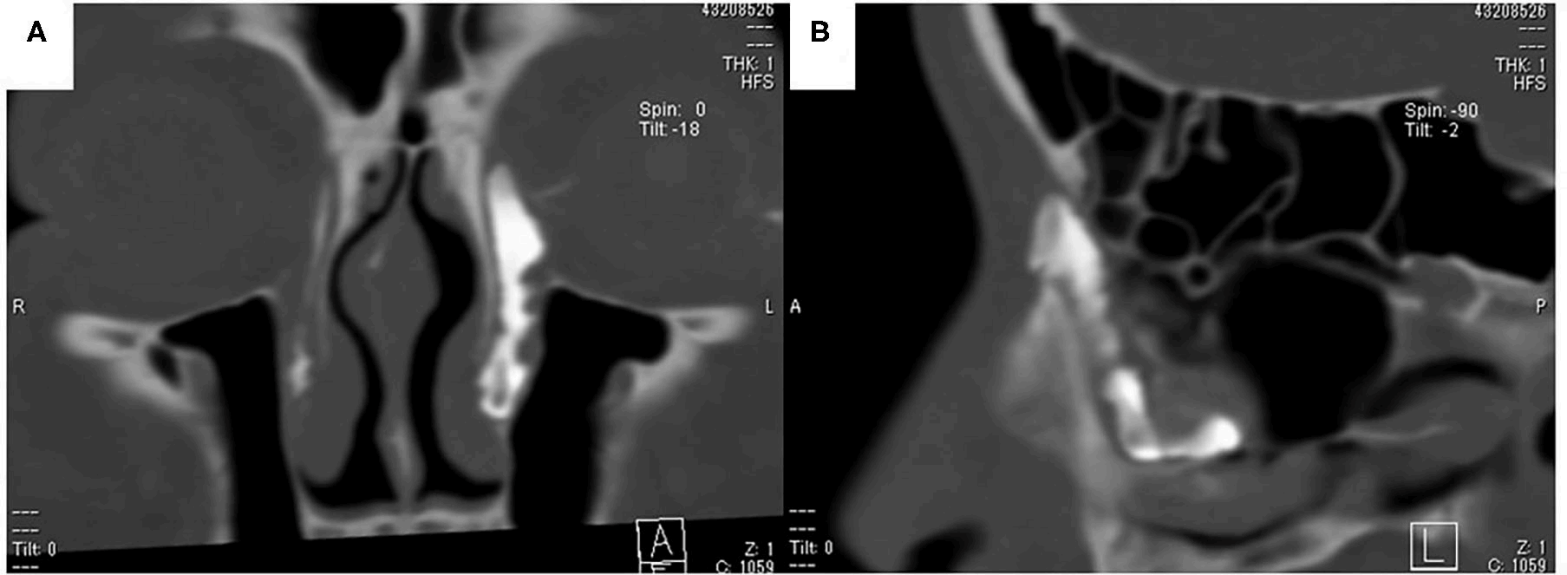
CT-dacrocystographic findings. **(A)** Coronal view: contrast medium was vertically located from the lacrimal sac to the orifice. **(B)** Sagittal view: contrast medium accumulated posteriorly from the lacrimal orifice.

**Figure 4:**
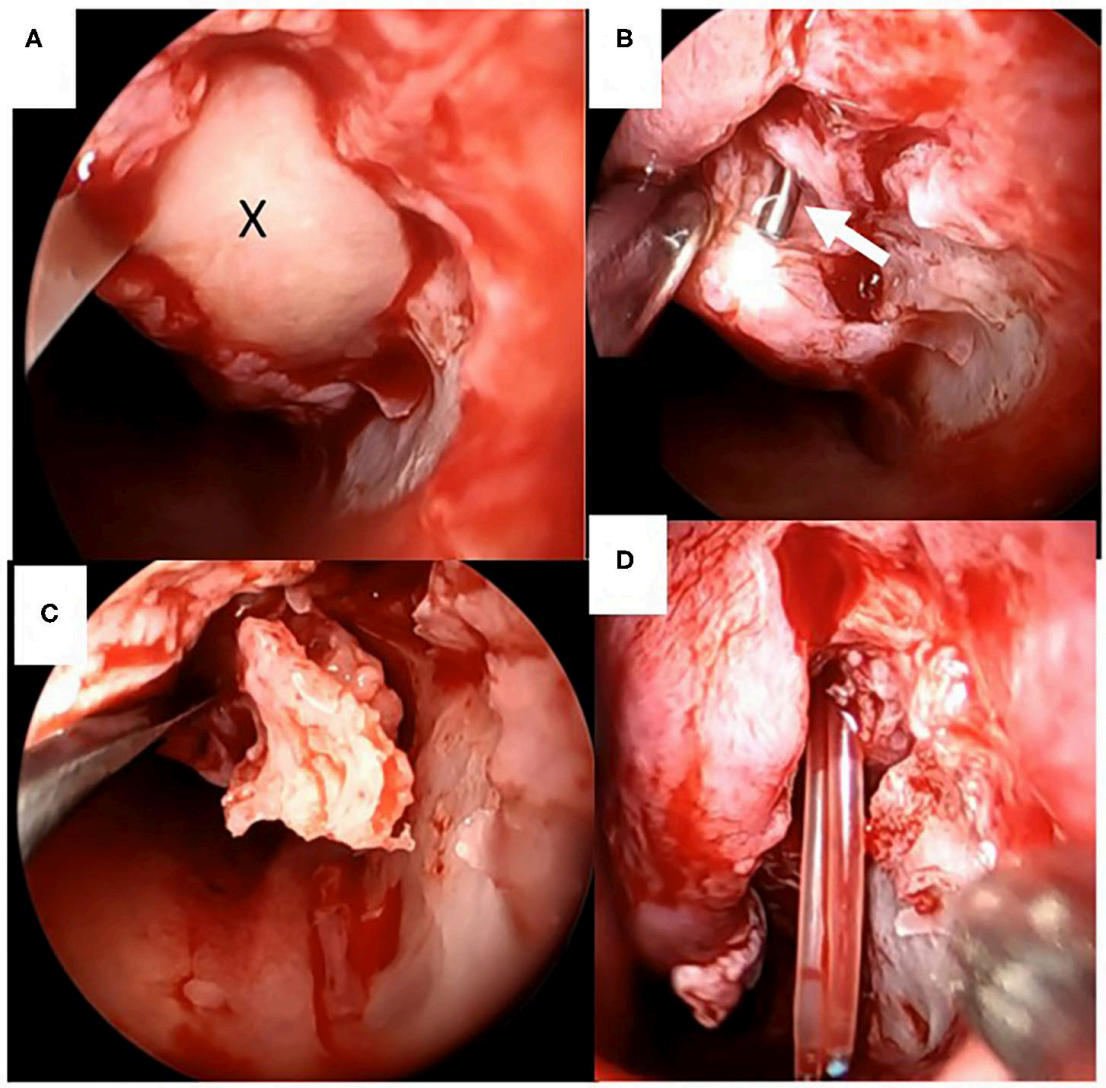
Intraoperative endoscopic findings of marsupialization of the cystic lesion to the inferior meatus. **(A)** Drainage of purulent discharge (X) from the cystic lesion between the inferior turbinate and the lateral wall of the inferior meatus. **(B)** Tip of the lacrimal probe indicates the nasolacrimal orifice (white arrowhead). **(C)** The vertical bone of the inferior turbinate was separated from the surrounding tissue. **(D)** Silastic lacrimal intubation tubes were retrieved endonasally from the nasolacrimal orifice.

**Figure 5:**
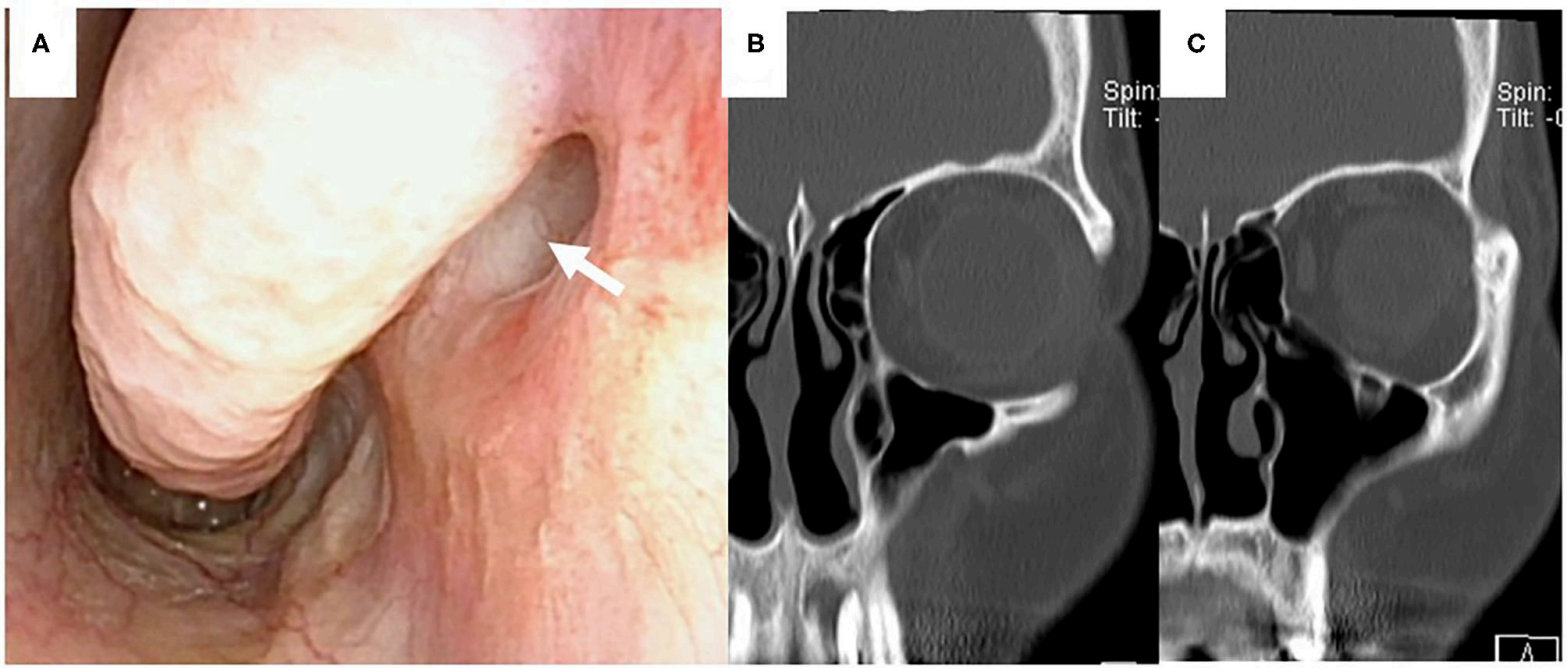
Post-operative findings 6 months after the operation. **(A)** Fiberscopic findings showed that the nasolacrimal orifice could be observed in the inferior meatus (white arrow). **(B,C)** Coronal CT scan findings showed the inferior meatus and patency of the nasolacrimal duct and orifice.

Flexible endonasal fiberscopic examination showed bilateral inferior turbinate hypertrophy. In addition, the anterior part of the left inferior nasal meatus had disappeared because the lateral portion of the inferior turbinate was adhering to the lateral wall of the inferior meatus (Figure 1). Coronal computed tomography (CT) scan showed enlargement of the left lacrimal duct (Figure 2A), and an axial CT scan showed the nasolacrimal orifice to be covered by soft tissue of the inferior turbinate (Figure 2B). Coronal CT-dacrocystography (CT-DCG) also showed enlargement of the left lacrimal duct from the lacrimal sac to the nasolacrimal orifice (Figure 3A). In addition, sagittal CT-DCG revealed that an accumulation of contrast medium was extending posteriorly from the nasolacrimal orifice (Figure 3B). These findings suggested that the synechia occurring post-operatively following the previous inferior turbinate surgery had created a cystic lesion that stored lacrimal fluid between the inferior turbinate and the lateral wall of the internal meatus. To open the nasolacrimal orifice to the inferior meatus, we performed endoscopic marsupialization of the cystic lesion to the inferior meatus. First, the inferior turbinate was separated medially from the adherent lateral nasal wall. After the mucous membranes at the attachment of the inferior turbinate to the lateral nasal wall were cut, the adhesion of the deformed vertical bone of the inferior turbinate to the lateral nasal wall was located. We exfoliated it from front to back to recreate an inferior meatus. For easy recognition of the location of the cystic lesion, a lacrimal passage endoscope was introduced from the inferior punctum of the lower eye lid to the cystic lesion by an ophthalmologist. The cystic lesion was transilluminated, and the wall of the cystic lesion was incised and widely opened with a mini-sickle knife. At this time, purulent discharge could be drained from the cystic lesion (Figure 4A). The tip of the lacrimal passage endoscope indicated the location of the nasolacrimal orifice as shown in Figure 4B, suggesting that the nasolacrimal drainage system, consisting of the puncta of the eyelid, the canaliculi, the lacrimal sac and the lacrimal duct and orifice, were clearly patent. After removal of the deformed vertical bone of the inferior turbinate (Figure 4C), a microdebrider blade was used to remove hypertrophic submucosal tissue from the lateral aspect of the inferior turbinate, which helped the remaining mucosa to roll upon itself to cover all raw surfaces. To prevent restenosis, silastic lacrimal intubation tubes (LACRIFAST, Kaneka Medix Corporation, Osaka, Japan) were placed through the upper and lower puncta, retrieved endonasally from the nasolacrimal orifice (Figure 4D), and then were left in place for 3 months after the operation. To prevent re-adhesion, we used alginate wound dressings (Sorbsan, ALCARE, Tokyo, Japan) placed over the rolled mucosal flap reforming the turbinate to keep it in place during the operation. Antibiotics were administered to manage the biofilm in the area of the surgery. The patient was instructed to perform nasal irrigation by herself every day at home. In addition, she consulted us periodically to manage the crust in the inferior meatus and to confirm patency of the lacrimal orifice via the flexible endonasal fiberscope. The patient has not experienced a recurrence of epiphora or purulent discharge from her left eye. Three months after the operation, we removed the lacrimal intubation tubes because the lacrimal orifice was widely patent. By 6 months after the operation, repeat flexible endonasal fiberscopy showed preservation of the left inferior meatus and patency of the left nasolacrimal orifice (Figure 5A). In addition, a coronal CT scan showed that the nasolacrimal duct was widely patent (Figures 5B,C).

## Discussion

We reported the first case of post-operative NLDSO following submucosal resection of the inferior turbinate. Because of its anatomical characteristics (1), the lacrimal drainage system can be injured with respect to functional endoscopic sinus surgery and several other otolaryngologic surgeries (2–4). Lacrimal secretions are initially collected by the superior and inferior puncta of the upper and lower eyelids. The canaliculi extends from the punctum to the lacrimal sac. The sac courses inferiorly and narrows to form the nasolacrimal duct, which ends in the lacrimal orifice at the lateral wall of the inferior meatus, ~3–4 cm from the anterior nares (5). In the endonasal procedures reported in 1990's (2, 3), the lacrimal orifice was most likely to be damaged. Osguthorpe and Calcaterra reported six cases of NLDSO after inferior meatus antrostomy (2). Meyers and Hawes also described four cases of NLDSO after the same operation (3). In one of the four cases, both septoplasty and turbinectomy were also performed. Upon introduction of the endoscopic sinus surgery (ESS) technique (4), the most likely sites of injury to the nasolacrimal drainage system were reported to be to the lacrimal duct during enlargement of the middle meatus antrostomy anteriorly with backbiting forceps or to the lacrimal sac during the initial lateral nasal wall incision with a sickle knife. Synechiae of the sinonasal cavity have traditionally been considered a complication of sinus surgery, and synechia formation in the middle meatus is the most common complication of ESS (6). Synechiae between the medial faces of the middle and/or inferior turbinates and the septum are called medial synechiae, whereas the term “lateral synechia” is used for an adhesion between the lateral face of the middle turbinate and the lateral nasal wall (7). In our investigation of the literature on PubMed and Ichushi (a Japanese retrieval system), we could find no cases of NLDSO with the exception of that following inferior and middle meatus antrostomy.

At least 13 surgical techniques have been used over the past 130 years to treat hypertrophy of the inferior turbinate (8). Synechiae to the nasal septum are one of the complications reported in the previous studies. In partial and total inferior turbinectomy, King and Marby (9) reported that synechiae occurred in 5.9–12.0% of the patients. In powered inferior turbinoplasty, Friedman et al. (10) reported that a synechia was found in 5% of their patients. Hol and Huizing (8) pointed out that synechia may occur following laser surgery of the turbinate. In our patient, submucosal resection of the inferior turbinate was performed at the initial surgery, and post-operative synechiae caused the adhesion between the inferior turbinate and the lateral nasal wall of the inferior meatus. Our investigation of the literature on PubMed and Ichushi found no cases of post-operative synechiae-induced NLDSO caused by inferior turbinate surgery including submucosal resection of the inferior turbinate. Therefore, we thought our case to be the first case report.

Epiphora secondary to NLDSO is usually treated with a dacryocystorhinostomy (DCR) performed via an open or endonasal approach (4). Recently, powered endoscopic DCR has become widely accepted (11). In the management of congenital NLDSO, the endonasal surgical approach through the inferior meatus was reported in addition to DCR (12). Korkmaz et al. (13) noted that an endoscopic incision was performed to perforate the preserved Hasner membrane of the lacrimal orifice. Dogan et al. (14) performed endoscopic marsupialization of congenital nasolacrimal duct cysts. In adults, Eloy et al. (15) reported three patients with cystic dilatation of the distal end of the nasolacrimal duct beneath the inferior turbinate. They performed DCR in one patient with a very small cyst beneath the inferior turbinate but performed endoscopic marsupialization in the other two patients with a cyst significant enough to apply the microdebrider from the inferior meatus to the lesion with ease and safety. They concluded that endoscopic marsupialization of the nasal expansion is the preferred choice. In our patient, CT-DCG showed enlargement of the lacrimal duct from the sac to the orifice, and the accumulation of contrast medium extended from the orifice posteriorly and horizontally. We thought that the lacrimal duct itself was not injured during the initial procedure but rather that adhesions had formed around Hasner's valve of the lacrimal orifice and also that this accumulation was likely to be a significant cystic lesion continuing from the orifice. We performed endoscopic marsupialization of the cystic lesion first instead of DCR, which is usually required to cure a more proximal nasolacrimal duct occlusion. Then, to open the lacrimal orifice into the inferior meatus, we followed with powered inferior turbinoplasty. Six months after the operation, both endonasal fiberscopic examination and coronal CT scan showed the left inferior meatus and nasolacrimal orifice to be widely patent. Based on our experience, we thought that the NLDSO was caused by synechiae, which are a complication of insufficient inferior turbinate surgery.

## Conclusion

We report the first case of post-operative NLDSO following inferior turbinate surgery. CT-DCG showed patency of the lacrimal duct from the sac to the orifice, and significant cystic accumulation of contrast medium extended from the orifice, suggesting that the synechia occurring post-operatively following the previous inferior turbinate surgery had created a cystic lesion that stored lacrimal fluid between the inferior turbinate and the lateral wall of the internal meatus. We performed endoscopic marsupialization of the cystic lesion and repeated the inferior turbinoplasty. Nasal endoscopy and CT-DCG are useful to diagnose the location of a NLDSO. Otorhinolaryngologists should be aware that surgery may lead to the formation of a NLDSO and that endoscopic marsupialization is a curative treatment for these distal-end NLDSOs. Based on the findings in our patient, NLDSO might be caused by synechiae, which are a complication of inferior turbinate surgery.

## Data Availability Statement

The raw data supporting the conclusions of this article will be made available by the authors, without undue reservation.

## Ethics Statement

Ethical review and approval was not required for the study on human participants in accordance with the local legislation and institutional requirements. The patients/participants provided their written informed consent to participate in this study. Written informed consent was obtained from the individual(s) for the publication of any potentially identifiable images or data included in this article.

## Author Contributions

HTac, HS, MF, and HTak: conception and design. HTac and HS: literature search and obtaining of images. HS and HTak: wrote the article. All authors: critical revision and final approval of the article.

## Conflict of Interest

The authors declare that the research was conducted in the absence of any commercial or financial relationships that could be construed as a potential conflict of interest.

## References

[1] Della Rocca DA Ahmad S Preechawi P Schaefer SD Della Rocca RC. Nasolacrimal system injuries. In: Weber RK, Keerl R, Schaefer SD, Della Rocca RC, editors. Atlas of Lacrimal Surgery. New York, NY: Springer (2007). p. 91–103. 10.1007/978-3-540-68215-8_9

[2] Osguthorpe JD Calcaterra TC. Nasolacrimal obstruction after maxillary sinus and rhinoplastic surgery. Arch Otolaryngol. (1979) 105:264–66. 10.1001/archotol.1979.00790170034009 435149

[3] Meyer AD Hawes MJ. Nasolacrimal obstruction after inferior meatus nasal antrostomy. Arch Otolaryngol Head Neck Surg. (1991) 117:208–11. 10.1001/archotol.1991.01870140096015 1991066

[4] Cohen NA Antunes MB Morgenstern KE. Prevention and management of lacrimal duct injury. Otolaryngol Clin North Am. (2010) 43:781–8. 10.1016/j.otc.2010.04.005 20599082

[5] Shaeffer JP Types of ostia nasolacrimalia in man and their genetic significance. Am J Anat. (1912) 13:183–92. 10.1002/aja.1000130208

[6] Manji J Habib A Amanian AA Alsalen S Thamboo A Javer AR. Potential risk factors associated with the development of synechiae following functional endoscopic sinus surgery. Eur Arch Oto-Rhino-Laryngol. (2018) 275:1175–81. 10.1007/s00405-018-4936-1 29546557

[7] Mantovani M Rinaldi V Torretta S Sigismund PE Cappadona M Minetti A. The dragonfly splint: a new disposable device designed to prevent both medial and lateral turbinate synechiae after sinonasal surgery. J Craniofac Surg. (2014) 25:547–50. 10.1097/SCS.0000000000000388 24448524

[8] Hol MK Huizing EH. Treatment of inferior turbinate pathology: a review and critical evaluation of the different techniques. Rhinology. (2000) 38:157–66. 11190749

[9] King HC Mabry RL A Practical Guide to Management of Nasal and Sinus Disorders. New York, NY: Thieme Medical Publishers, Inc (1993). p. 94–118.

[10] Friedman M Tanyeri H Lim J Landsberg R Caldarelli D. A safe, alternative technique for inferior turbinate reduction. Laryngoscope. (1999) 109:1834–7. 10.1097/00005537-199911000-00021 10569417

[11] Wormald PJ Powered endoscopic dacryocystorhinostomy. In: Wormald PJ, editor. Endoscopic Sinus Surgery. Anatomy, Three-Dimensional Reconstruction, and Surgical Technique. 3rd ed New York, NY; Stuttgart: Thieme (2013). p. 136–62. 10.1055/b-0034-74899

[12] Avram E. Insights in the treatment of congenital nasolacrimal duct obstruction. Rom J Ophthalmol. (2017) 61:101–6. 10.22336/rjo.2017.19 29450381 PMC5710016

[13] Korkmaz H Korkmaz M Karakahya RH Serhath M. Endoscopic intranasal surgery for congenital duct obstruction–a new approach. Int J Pediatr Otorhinolaryngol. (2013) 77:918–21. 10.1016/j.ijporl.2013.03.005 23541294

[14] Dogan E Yüksel NG Ecevit MC Yaman A Berk AT Sütay S. Microdebrider assisted endoscopic marsupialization of congenital intranasal nasolacrimal duct cysts. Int J Pediatr Otorhinolaryngol. (2012) 76:488–91. 10.1016/j.ijporl.2011.12.031 22277269

[15] Eloy P Poirrier AL Nicoli T Marlair C Delahaut G Leruth E. Cystic dilatation of the distal end of the nasolacrimal duct: underrated cause of epiphora in adults and its endoscopic treatment. Rhinology. (2012) 50:436–41. 10.4193/Rhin12.067 23181256

